# Using nutrition to help recovery from infections

**DOI:** 10.1097/MOG.0000000000001068

**Published:** 2024-11-20

**Authors:** Ines B. Moura, Anthony M. Buckley

**Affiliations:** a Leeds Institute of Medical Research, Faculty of Medicine and Health, University of Leeds; b Microbiome and Nutritional Sciences Group, School of Food Science and Nutrition, University of Leeds, Woodhouse Lane, Leeds, UK

**Keywords:** antibiotic, diet, gut microbiota, healthy ageing, resilience

## Abstract

**Purpose of review:**

Antibiotics are a cornerstone of modern medicine, but antibiotic consumption can have depleting effects on the gut microbiota, potentially leading to gastrointestinal symptoms and other diseases, namely *Clostridioides difficile* infection. Because nutrition is a major driver of gut microbiota diversity and function, here we explore the current evidence on the potential of diets in alleviate the deleterious effects of antibiotics consumed during infections.

**Recent findings:**

Beneficial nutrients can enhance the symbiotic effect of the gut microbiota with the host, supporting anti-inflammatory responses and maintaining tight junction integrity. Short-chain fatty acids have been shown to positively affect the immune response, reducing the severity of *C. difficile* infection, whereas high-fibre diets have been shown to promote faster recovery of the gut microbiota after antibiotic therapy.

**Summary:**

The role of nutrition during infection is gaining momentum, with key findings exploring the effect of some nutrients in limiting the severity of infections and helping the microbiota recover from antibiotic-induced dysbiosis. Although this field is in its infancy, these findings open the possibility of personalised nutrition as a way of restoring microbiome diversity. But more work is needed to identify the most effective types and combinations of nutrients to achieve this.

## INTRODUCTION

The gut microbiota is a complex community of microorganisms, mostly bacteria, that live in the human intestine in a synergistic relationship with the host and its immune system [1]. Each person's microbiome results of environmental exposures and host genetics, forming a unique composition and diversity of microbes essential for nutrient digestion. The adult microbiota remains relatively stable throughout adult life, being largely characterized by Firmicutes (including genera *Lactobacillus*, *Clostridium*, *Enterococcus*, *Ruminococcus*, *Faecalibacterium* and *Roseburia*), Bacteroidetes (genera *Bacteroides* and *Prevotella*) and Actinobacteria (mainly genus *Bifidobacterium*) [2,3]. As adults, diet is the main driver of microbiota changes, impacting diversity and structure, and consequently its function [1,4]. Microbially produced compounds resulting of nutrient enzymatic metabolization have a crucial role in health and disease [5]. An imbalance of the gut microbiota due to poor diet, or dysbiosis, has been associated with several chronic and metabolic disorders, including obesity, type 2 diabetes, gastrointestinal, cardiovascular and respiratory diseases [4,6–11]. However, the main cause of microbial dysbiosis is associated with the use of xenobiotics, particularly broad-spectrum antibiotics, as their untargeted action potentiates the deleterious effect on the commensal gut microbes involved in digestion [12–14].

Here, we will discuss the interaction between host health and diet, particularly in the face of infection. 

**Box 1:**
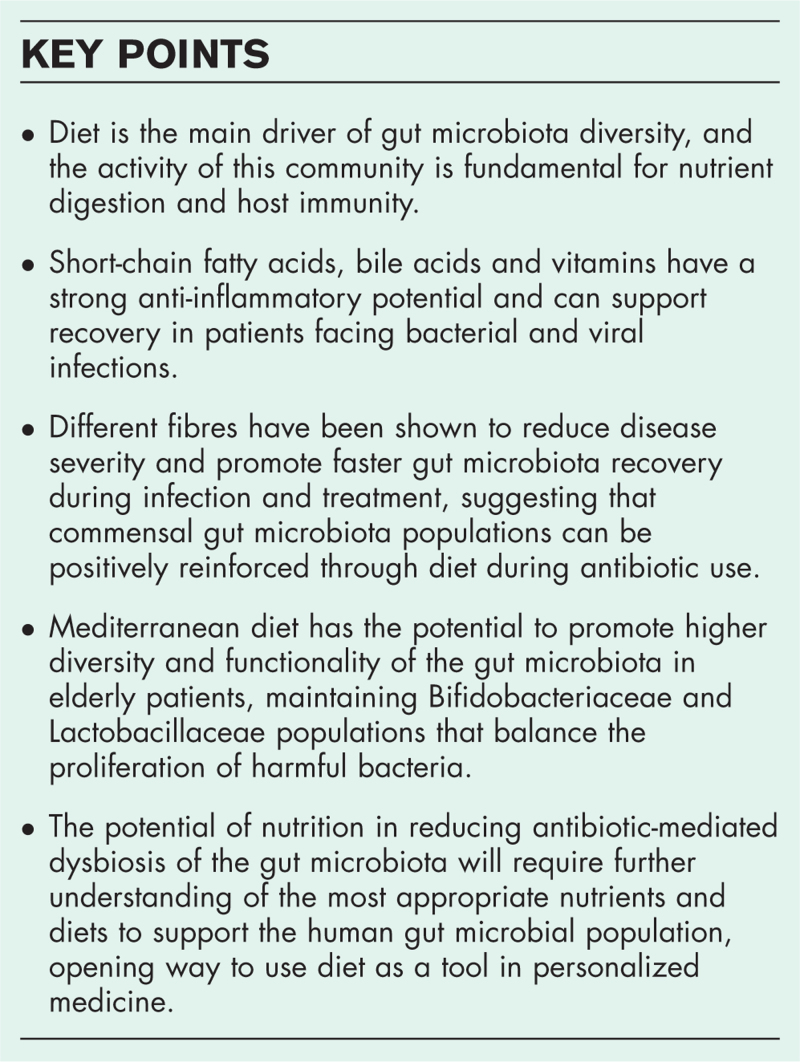
no caption available

## NUTRIENT METABOLISM IN HEALTH AND DISEASE

Given the prevalence of immune cells in the gastrointestinal track, the enzymatic capabilities of the gut microbiota in nutrient digestion and absorption have an impact in immune regulation and host physiology [15^▪▪^,^16^^▪▪^]. Microbial activity in the intestine is responsible for producing or modifying nutrients such as short-chain fatty acids (SCFAs) or bile acids; these biproducts of digestion have an impact on human health [16^▪▪^,^17^,18^▪^]. SCFAs are produced via fibre fermentation by Firmicutes and Bacteroidetes and their prebiotic properties are evidenced by supporting gut microbiota diversity [15^▪▪^,^16^^▪▪^,^17^]. Butyrate, acetate and propionate make for nearly 90% of the SCFAs present in the human colon. These compounds have been associated with host health through immunoregulation, maintaining the integrity of the intestine epithelial barrier and regulating gene expression [15^▪▪^,^16^^▪▪^,^17^,18^▪^,^19^]. Low production of SCFAs, such as butyrate, derived of the low prevalence of relevant gut bacteria (e.g. *Bacteroides* and *Faecalibacterium*), can have a systemic effect contributing to obstructive sleep apnoea [20] and an increased risk of colitis associated cancer [21]. Conversely, bile acids are produced in the liver and released in the intestinal lumen where they can be modified by bacteria to produce secondary and tertiary bile acids. These molecules act as emulsifiers of lipids and fat-soluble vitamins, facilitating their assimilation [12,22]. Investigating type 2 diabetes, Wang *et al.*[15^▪▪^] reported decreased bile acid production and abnormal lipid metabolism in mouse models. Diseased animals also showed a lower abundance of SCFAs-producing bacteria, compared with the study control group, potentially explaining the increased inflammation observed in patients with diabetes [15^▪▪^].

Furthermore, diversification of dietary nutrients supports a greater range of gut microbial species, with a higher microbial diversity associated with foods/nutrients with low inflammatory or anti-inflammatory properties [1,5,23,24]. For instance, polyphenols (e.g. tannins, phenolic acid, flavonoids) can reduce oxidative stress, preventing damage to DNA and cell components, and reducing lipid accumulation associated with ageing-related diseases, namely degenerative diseases, osteoporosis and cancer [2,25,26].

Human studies consuming high fibres, polyphenols, unsaturated fats and plant proteins, observed a higher microbial diversity compared to those on a high-sugar/high-saturated fat diet [19]. This is reflective of a [mostly] plant-based Mediterranean diet, which involves high intake of fruits, vegetables, legumes, nuts and cereals, and moderate consumption of fish and olive oil [24,27,28]. These foods contain high levels of beneficial nutrients linked to better diet quality, reduced diseases and lower chronic inflammation [5,28,29]. Mediterranean diet has been reported to support microbiome diversity and stability, leading to increased levels of Bifidobacteriaceae, Lactobacillaceae, Lachnospiraceae, Bacteroidaceae, and Prevotellaceae, as well as a decrease of Enterobacteriaceae [25,27,30]. The positive effects of a Mediterranean-type diet have also been shown to improve clinical outcomes in patients with cardiovascular disease [24,31], and to reduce intestinal symptoms and improve quality of life in patients with ulcerative colitis [29].

Diet has a crucial synergistic effect with the intestinal microbiota, modulating its structure and function and promoting the microbial diversity required to support effective digestion and immunity. Thus, changes in nutrient metabolism associated with dysbiosis can negatively impact human health.

## IMPACT OF DIET ON MICROBIOME RESILIENCE DURING INFECTIONS

Given the anti-inflammatory potential of certain foods, there is a strong potential to use nutrition as prebiotic, to reduce the risk of disease or support recovery in patients facing bacterial and viral infections [5]. Diet supplementation with the SCFAs propionate reportedly modulated the gut microbiota composition of HIV patients, namely through an increase of Ruminococcaceae and Bacteroidaceae. Ingestion of propionate was overall associated with a higher quality of life in HIV-positive patients, leading to reduced symptoms of noninfectious diarrhoea, and an increase in anti-inflammatory immune responses [18^▪^].

Macro and micronutrients such as vitamins, minerals and other nutraceuticals can also positively modulate the immune system aiding the immune response to infections. A prospective cohort study found that children with coronavirus disease 2019 (COVID-19) infection have significantly decreased serum levels of zinc and vitamin D [32]. This has been further confirmed in adults, where vitamin D supplementation alongside vaccination for COVID-19, significantly increased the levels of immunoglobulin G in symptomatic patients and reduced gastrointestinal symptoms by 44% following a second dose of the vaccine [33].

The impact of diet can also extend to disease severity and outcome, as seen in mouse models of *Clostridoides difficile* infection, where a high-fat/low fibre diet showed increased toxin levels and mortality, compared with a low-fat/low-fibre diet. A higher fat intake led to changes in the gut microbiota, resulting in increased production of primary bile acids associated with *C. difficile* spore germination (taurocholic acid and cholic acid), whereas production of CDI-suppressing secondary bile acids was reduced [7]. Although a protective effect of fibre was not found in that study, other murine models of CDI have observed lower inflammation and less toxin-mediated tissue damage in the intestine of mice when their diet was supplemented with pectin. These observations were supported by a higher diversity of the mice microbiota alongside a higher abundance of *Lachnospiraceae* and *Blautia* populations, known fermenters of fibres into CDI-inhibiting secondary SCFAs, such as chenodeoxycholic acid [34^▪▪^]. The role of dietary sugar trehalose in CDI induction has also been a point of research, with studies of the human microbiota using an in-vitro triple-stage model, consistently showing trehalose was not associated with CDI induction. Compared with exposure to glucose or isotonic saline, the colonic microbiota metabolizing trehalose showed enhanced abundance of *Finegoldia*, *Faecalibacterium* and *Oscillospira*, and reduced levels of *Klebsiella* and *Clostridium* spp. [35].

These studies suggest that diet and nutrient supplementation can be powerful tools in reducing inflammation and improving recovery during infections, underlining a potential complementary role between prebiotics and the currently favoured route of probiotics or microbiota replacement therapies.

The potential of diet in supporting microbiota resilience is of particular relevance during bacterial infections, which generally require one or multiple courses of antibiotic treatment. A proportion of any orally prescribed antibiotics will reach the colon, impacting the microbial populations via bactericidal or bacteriostatic effect [7,13]. Broad-spectrum antibiotics such as clindamycin, azithromycin or amoxicillin can impact the human gut microbiota extensively and persistently, depleting commensal populations of Bifidobacteriaceae, Lactobacillaceae, Bacteroidaceae and Lachnospiraceae [13,35,36]. These off-target deleterious effects can create the conditions for pathobionts such as *E. coli*, *Klebsiella* or *Enterococcus* to overgrow, leading to subsequent infections and requiring further antibiotic treatment. This debilitating cycle supports the spread of antibiotic-resistant genes and is often accompanied by gastrointestinal symptoms, such as vomiting and diarrhoea, which lessen patients’ quality of life [13,14,35,36].

Given the reported prebiotic potential of certain nutrients (vitamins, fibres, polyphenols, etc.), it is possible that commensal gut microbiota populations can be positively reinforced through diet during antibiotic use. By maintaining key metabolic pathways involved in SCFAs and bile acid production, the detrimental effects of antibiotics could be limited, breaking the cycle of successive infections.

This hypothesis is reinforced by recent murine studies, where diet supplementation with seven different plant fibres, including pectin and inulin, resulted in a less dysbiotic microbiota profile following amoxicillin treatment. Fibre supplementation also promoted a faster recovery of the microbiota, increased the diversity of bacterial populations after antibiotic treatment and resulted in lower oxidoreduction potential [37]. Similarly, another study observed that a low fibre diet in mice can contribute to an extended antibiotic-mediated dysbiosis, with a slower recovery of the gut microbiota [7]. With the decline of different populations, the microbial metabolic pathways that contribute to host-microbial homeostasis were damaged, leading to increased oxygen levels in the lumen and inhibiting the activity of secondary bile acid producers such as *Clostridium scindens*[7].

Overall, dietary choices can affect the gut microbiota structure and metabolism, supporting the hypothesis that changes in diet may affect the occurrence and development of infections. Current data also suggest that targeted nutrition has the potential to increase microbiota resilience in the presence of infections and throughout antibiotic treatment. Still, further work is required to fully understand if these positive effects are seen across different classes of antibiotics and at different stages of dietary intervention. Importantly, there is limited information on the impact of diet during infections on the human microbiota, as most studies use murine models as the host, and these have a different microbiota composition to humans [38].

## DIET AND HEALTHY AGEING

As we age, the lifetime accumulation of assaults to our gut microbiome causes a decline in diversity and functionality, which is associated with an increased risk to several intestinal and extra-intestinal diseases, for example cardiovascular disease [11,39].

There is increasing evidence that the gut microbiota composition can function as a marker of healthy ageing, with association to frailty and disease severity [40–42]. As we age, frailty increases [43] and so does the frequency of antibiotic use [14], either as treatment for infections or as prophylaxis before elective surgery, increasing the potential for microbiota disruption and opportunistic infections.

Colonic microbial composition remains relatively stable throughout adult life but, in older age (>65 years), depletion of certain populations such as *Bacteroides*, *Prevotella* or *Roseburia* and variations in amino acid metabolism [40,44,45], suggest a compositional shift in the balance between beneficial and potentially harmful bacteria that correlates with health decline, an indication that gut microbiota patterns could not only inform but also contribute to the host life expectancy [1,40,45].

A positive association between a healthy/balanced diet and delayed/better ageing can be established through nutrient modulation of the gut microbiota, resulting in less inflammation and/or oxidative stress [23], and lower risk of cardiovascular disease [39]. Profiling the gut microbiota of a group of centenarians and nonagenarians from Italy that followed a Mediterranean diet, researchers reported a positive association between longer life and the microbiota composition. Notably, these elderly individuals showed good diversity and abundance of Bifidobacteriaceae and Lactobacillaceae, compared to younger controls. These populations are known for their interaction with anti-inflammatory and anti-oxidant microbial processes, and are often depleted in individuals who do not live until such later years, suggesting that protecting/promoting their prevalence in the elderly microbiota could balance the pathogenic potential of pathobionts [46^▪▪^].

Other studies in humans [47^▪^] and in murine models [48] have reported beneficial effects of fibre-based prebiotics (inulin and oligofructose) and polyphenols (genistein) in reducing frailty and improving lifespan, by modulating the elderly gut microbiota composition. Notably, the supplementation with fibres led to an increase in protein digestion, which supported better health in later years. This is consistent with previous observations of individuals over 67 years of age showing lower levels of physiological dysregulation when ingesting higher protein and vitamin E, whereas high levels of carbohydrates were associated with poor health [42].

## CONCLUSION

Current data suggest that diet and gut microbiota are closed related with pathways of inflammation, lipid digestion and SCFA regulation; however, much remains unclear about the patterns of gut microbiota ageing and how to delay or revert them. The limitations inherent to observational and animal studies restrict our understanding into the causation of microbiome changes during disease or ageing, with more intervention studies required. Improved insights into our understanding of the microbial pathways through which dietary interventions can be used to minimize antibiotic-mediated dysbiosis will open new perspectives to support healthy ageing.

## Acknowledgements

*None.*

### Financial support and sponsorship

*None.*

### Conflicts of interest

*There are no conflicts of interest.*

## REFERENCES AND RECOMMENDED READING

Papers of particular interest, published within the annual period of review, have been highlighted as:

▪ of special interest
▪▪ of outstanding interest
